# Microfused essential oil supplementation remodels the plasma metabolome and identifies candidate biomarkers associated with growth performance in Katahdin ewes

**DOI:** 10.3389/fvets.2026.1941177

**Published:** 2026-09-09

**Authors:** Yariana Woody, Oluwatobi Fijabi, Brou Kouakou, Thomas Terrill, Yarahy Leal, Samanthia Johnson, Ibukun M. Ogunade, Godstime Taiwo

**Affiliations:** 1 College of Agriculture, Family and Consumer Sciences, Fort Valley State University, Fort Valley, GA, United States; 2 Department of Animal and Range Sciences, New Mexico State University, Las Cruces, NM, United States; 3 Department of Agriculture, Illinois State University, Normal, IL, United States; 4 Division of Animal Science, West Virginia University, Morgantown, WV, United States

**Keywords:** growth performance, Katahdin ewes, microfused essential oil blend, phytogenic feed additive, plasma biomarkers, untargeted metabolomics

## Abstract

This study investigated plasma metabolic responses to microfused essential oil blend (MEO) supplementation and identified candidate biomarkers associated with growth performance in Katahdin ewes. Eighteen Katahdin ewes (26.34 kg) were randomly assigned to a basal diet (CON; *n* = 9) or the basal diet supplemented with 4 g/head/day of a microfused essential oil blend containing oregano (*Origanum vulgare*), cinnamon (*Cinnamomum* spp.), and white thyme (*Thymus vulgaris*) (MEO; *n* = 9) for 35 days. Growth performance was evaluated, and plasma metabolomic profiling was performed using chemical isotope labeling liquid chromatography–mass spectrometry. MEO supplementation increased final body weight, total weight gain, and average daily gain (ADG) while reducing feed:gain (*p* < 0.05). Untargeted metabolomics identified 12 differentially abundant metabolites (FDR < 0.05), predominantly showing lower circulating concentrations in MEO-supplemented ewes. Pathway analysis revealed significant enrichment of linoleic acid metabolism, α-linolenic acid metabolism, and biosynthesis of unsaturated fatty acids (*p* ≤ 0.05). Receiver operating characteristic (ROC) analysis identified four candidate plasma biomarkers: *γ*-amino-*γ*-cyanobutanoic acid, Nocardicin A/Isonocardicin A, 2-propylmalic acid, and isoleucine that discriminated MEO from CON ewes (AUC > 0.85), while a combined four-metabolite panel achieved an AUC of 0.943 (95% CI: 0.778 to 1.000). Correlation and regression analyses further identified *γ*-amino-*γ*-cyanobutanoic acid and isoleucine as independent predictors of ADG and feed efficiency. Microfused essential oil supplementation improved growth performance and induced coordinated remodeling of the plasma metabolome in Katahdin ewes. The identified metabolic pathways and candidate biomarkers provide mechanistic insight into the physiological responses to phytogenic supplementation and represent promising targets for monitoring nutritional interventions in sheep.

## Introduction

Feed accounts for more than 70% of livestock production costs (1), making nutritional strategies that improve feed efficiency a priority in ruminant systems (2). Rising antimicrobial resistance in food animal production has further accelerated the search for evidence based, non-antimicrobial alternatives that can sustain productivity and safeguard health in small ruminant systems. Consequently, phytogenic feed additives have gained considerable attention as sustainable alternatives to antibiotics that can enhance animal performance while supporting environmental stewardship and consumer expectations (3).

Essential oils (EOs) are plant-derived bioactive compounds with well-documented antimicrobial, antioxidant, anti-inflammatory, and digestive-modulating properties (4–6). Among the most widely investigated are oregano (*Origanum vulgare*), cinnamon (*Cinnamomum* spp.), and white thyme (*Thymus vulgaris*), which have been associated with improvements in growth performance, feed efficiency, nutrient utilization, and oxidative status in ruminants (3, 7–10). Essential oils are highly volatile and susceptible to oxidative rancidity during feed processing and deterioration during passage through the gastrointestinal tract. This limits the biological efficiency for livestock production. To overcome these limitations, advanced delivery technologies such as microfusion and microencapsulation have been developed to enhance the stability, bioavailability, and controlled release of essential oil bioactive compounds within the gastrointestinal tract. Evaluation of protected essential oil formulations has previously shown improvements in rumen fermentation, nutrient utilization, intestinal integrity, antioxidant status, immune function, and growth performance compared to unprotected essential oils. These enhancements have been attributed to the protection of the bioactive compounds during transit time in the gastrointestinal tract rather than the endogenous biological potential of essential oils. Nevertheless, direct comparisons between microfused and conventional essential oils in ruminants remain scarce, highlighting the need to determine whether improved delivery method is translatable into measurable metabolic and productive responses (11, 12).

At the biochemical level, oregano and thyme deliver the monoterpenoid phenols carvacrol and thymol, which disrupt bacterial cell membranes and inhibit Gram-positive and Gram-negative pathogens through membrane permeabilization and interference with proton motive force (4, 9). Cinnamon derived cinnamaldehyde and eugenol act on microbial ATPase activity, downregulate quorum sensing, and modulate host inflammatory signaling through inhibition of NF-κB and MAPK cascades. Downstream of these direct antimicrobial and immunomodulatory actions, essential oil bioactives have been reported to enhance intestinal digestive enzyme activity, support mucosal barrier integrity, and increase systemic antioxidant capacity in ruminants (8, 13, 14), establishing a mechanistic basis for the improved performance, feed efficiency, and antioxidant status observed following phytogenic supplementation.

Although improvements in animal performance following essential oil supplementation have been widely reported, the systemic metabolic mechanisms underlying these responses remain poorly understood. Untargeted plasma metabolomics provides a comprehensive assessment of host metabolism by simultaneously identifying differentially abundant metabolites, perturbed biochemical pathways, and candidate biomarkers associated with dietary interventions (15–17). While rumen metabolomics provides valuable insight into microbial fermentation, plasma metabolomics captures the integrated metabolic responses of the rumen microbiome and host tissues, making it particularly suitable for identifying minimally invasive biomarkers associated with growth performance and feed efficiency. Previous studies have successfully used plasma metabolomics to identify biomarkers of feed efficiency and production traits in sheep (18, 19), demonstrating its value as a discovery platform for nutritional research.

To our knowledge, no previous study has comprehensively characterized the plasma metabolomic response to microfused essential oil supplementation in Katahdin ewes or identified candidate plasma biomarkers associated with growth performance and feed efficiency. Addressing this knowledge gap could improve our understanding of the mechanisms through which phytogenic feed additives influence animal physiology and facilitate the development of biomarkers for evaluating nutritional interventions.

Essential oils have been recognized for influencing systemic metabolism in ruminants via direct modulation of the ruminal ecosystem, where their bioactive molecules could alter microbial community structure, fermentation characteristics, and microbial metabolism. These alterations could inform the profile of volatile fatty acids and other microbial signaling molecules that are absorbed systemically for ruminant metabolism and tissue deposition. Consequently, changes induced by dietary supplementation in the plasma metabolome could signify a systems-level representation of the ruminant physiological metabolism. Because metabolic processes are closely linked with growth and feed efficiency, and phytogenic feed additives rapidly influence metabolic and systemic profiles (20, 21), we hypothesized that 35 days of supplementation with a microfused essential oil blend would alter the plasma metabolome of growing ewes, improve average daily gain and feed efficiency, and identify metabolite biomarkers associated with growth. Therefore, this study employed untargeted plasma metabolomics to characterize the metabolic response of Katahdin ewes to microfused essential oil supplementation and to identify candidate biomarkers and metabolic pathways associated with growth performance.

## Materials and methods

### Experimental design and animal husbandry

The experiment was approved by the Institutional Animal Care and Use Committee of Fort Valley State University (IACUC protocol number SU R-02-2025). Eighteen healthy purebred Katahdin ewes, a hair sheep breed (approximately 6 months of age; average BW = 26.34 kg) were individually penned and acclimated for 21 days prior to the start of the experimental period to minimize handling stress and establish baseline feed intake patterns. Ewes were housed in individual pens (1.2 m × 1.2 m, providing about 1.4 m^2^ of floor space per ewe) constructed from welded-wire panels within an enclosed, roofed metal barn, bedded with wood shavings, and each pen was fitted with a standard metal feeder and a plastic water trough supplied by an automatic float valve that provided continuous access to fresh water throughout the study. Following acclimation, ewes were stratified by initial body weight and randomly assigned to one of two dietary treatments (*n* = 9 per group): a control group (CON) receiving a commercially formulated basal diet (Godfrey’s Show and Grow Lamb Feed; minimum 16% CP, minimum 2.4% crude fat, maximum 13% crude fiber); or the basal diet supplemented with 4 g per head per day of Microfused^®^ EO 100 Mini Pellet (MEO; Ralco Inc., Marshall, MN, United States), a stabilized microfused essential oil blend (Strong Animals^®^ brand) containing oregano (*Origanum vulgare*), cinnamon (*Cinnamomum* spp.), and white thyme (*Thymus vulgaris*). According to the manufacturer label (Ralco Nutrition, Inc.; formula Q2612), the Microfused^®^ EO 100 Mini Pellet is labeled for ruminants, including sheep, at a continuous feeding rate of 4 g per 100 lb. of body weight for animals under 250 lb. At the mean initial body weight of the ewes (26.3 kg, approximately 58 lb), this corresponds to approximately 2.3 g per head per day, so the 4 g per head per day used in the present study provided approximately 1.7 times the weight-scaled label rate to ensure a robust and consistent bioactive exposure across animals. The 35 d supplementation period was selected as it approximates the standard essential oil trial window in growing ruminants (3, 13) and is sufficient to detect systemic metabolic responses to phytogenic supplementation while remaining within the growth phase of Katahdin ewes. The Microfused^®^ EO technology reduces essential oil droplet size to enhance direct contact with rumen and gut microbial targets, and the product is stabilized against oxidative and gastrointestinal degradation to provide sustained bioactive delivery under practical feeding conditions (11, 22). The exact concentrations of the phenolic bioactive constituents (carvacrol and thymol from oregano and white thyme; cinnamaldehyde from cinnamon) are proprietary to the manufacturer and were not disclosed by the supplier. As listed on the product label (formula Q2612), the pellet comprised processed grain by-products, calcium carbonate, diatomaceous earth (organic flow agent), vegetable oil, and organic hemicellulose extract as carriers, with the essential oil actives supplied as origanum (oregano) oil, white thyme oil, and cinnamaldehyde; the guaranteed analysis specified crude protein (minimum 10.89%), crude fat (minimum 3.90%), crude fiber (maximum 5.20%), lysine (minimum 0.40%), calcium (9.80 to 11.70%), phosphorus (minimum 0.60%), and potassium (minimum 0.60%). Because the additive was fed at only 4 g per head per day, its contribution to dietary nutrient supply (approximately 0.4 g of crude protein and 0.4 g of calcium per day) was negligible, and the observed responses are attributed to the essential oil bioactives rather than the pellet carrier. A limitation of this study is that the exact concentrations of the bioactive compounds in the microfused essential oil (MEO) formulation were not available because the product is a proprietary commercial formulation. Although the manufacturer disclosed the principal active ingredients, the undisclosed concentrations limit the exact reproducibility of the dietary treatment and complicate direct comparisons with other essential oil formulations. Nevertheless, the dietary inclusion rate, product source, and supplementation protocol are fully described to facilitate replication as closely as possible. Future studies evaluating MEO or similar commercial formulations would benefit from greater transparency regarding product composition, as disclosure of bioactive compound concentrations would improve mechanistic interpretation, reproducibility, and comparisons across studies. The additive was administered as a top dress hand mixed with the daily ration to ensure complete intake. Feed was offered daily at 4% of individual body weight and adjusted weekly, and orts were removed each morning before fresh feed delivery. Feed refusals were negligible and did not differ between treatments; because they were not quantified on a per animal basis, dry matter intake (DMI) was estimated as the amount of feed offered, which we acknowledge as a limitation of the intake and feed efficiency estimates. Animals were housed under identical conditions throughout the 35-day experimental period, with body weight and performance recorded weekly. Average daily gain (ADG, kg/day) was calculated as the difference between final and initial body weight divided by 35 days, and the feed:gain was calculated as total dry matter intake (kg) divided by total weight gain (kg).

### Blood sample collection and plasma preparation

Blood was collected by jugular venipuncture between 08:00 and 09:00 h, following a 12 h overnight fast, into 10 mL EDTA vacuum tubes (Vacutainer, Becton Dickinson, Franklin Lakes, NJ, United States), maintained on ice, and processed within 2 h of collection. Plasma was obtained by centrifugation at 3,000 × g for 15 min at 4 °C, transferred into labeled microcentrifuge tubes, and stored at −80 °C until metabolomic analysis.

### Sample preparation for plasma metabolomics profiling

In the present study, plasma samples collected on days 7, 14, 21, 28, and 35 from each animal were composited in equal aliquot before they were subjected to untargeted metabolomic analysis. This was done to generate a representative metabolic profile associated with a cumulative physiological response to MEO additive over 35-day feeding trial. We adopted this method because our objective was to identify molecular signatures associated with MEO supplementation and putative exploratory biomarkers linked to growth performance rather than characterize temporal metabolic changes in growing ewes. Metabolites from the composited samples were extracted using a methanol protein precipitation method as previously described (23).

### CIL/LC–MS based metabolomics analysis

In depth untargeted metabolome profiling of the extracted plasma was performed using a chemical isotope labeling (CIL) liquid chromatography mass spectrometry (LC–MS) based technique. The technique uses differential 12C/13C isotope labeling to derivatize metabolites based on their chemical functional groups (amines/phenols, carboxylic acids, and carbonyls), enabling comprehensive sub-metabolome coverage (24). Detailed descriptions of the metabolite labeling, sample normalization using liquid chromatography with ultraviolet detection (LC-UV) quantification of labeled metabolites, and LC–MS operating conditions and setup have been reported previously (24). Relative quantification of 12C/13C-labeled metabolites based on peak pair ratios was performed on a compact quadrupole time of flight mass spectrometer coupled to an ultra-high-performance liquid chromatography system. A total of 18 LC–MS data files were generated (9 MEO and 9 CON samples), with 3 quality control samples analyzed at regular intervals throughout the analytical run.

### Metabolite data processing and identification

All raw LC–MS data files were processed using IsoMS Pro 1.0 following the procedures described by Mung and Li (25). Briefly, 12C/13C peak pairs were extracted from each run by the IsoMS software. In this step, redundant pairs (those of adduct ions and dimers) and noise signals (having a singlet peak) were filtered out, retaining only the protonated ion of a peak pair for each true metabolite, and the peak intensity ratio was calculated for each peak pair. The IsoMS-Quant program was then used to determine the chromatographic peak ratio of each peak pair and to generate the final metabolite intensity table (26). Metabolite identification employed a three tier approach: in Tier 1, peak pairs were searched against the CIL metabolite library (containing 1,060 unique endogenous metabolites) based on accurate mass and retention time for positive identification; in Tier 2, a Linked Identity (LI) library containing over 2,000 metabolic pathway related metabolites extracted from the KEGG database was used for high confidence putative identification based on accurate mass and predicted retention time (27); in Tier 3, the MyCompoundID library was searched for additional putative identifications. A total of 8,669 peak pairs were detected across all samples. The 8,669 value refers to raw peak pairs (spectral features) rather than annotated metabolites; of these, 1,005 were annotated to known metabolites and 601 were retained after quality control and filtering. Identification confidence followed the Metabolomics Standards Initiative (MSI): Tier 1 matches (accurate mass and experimental retention time against the CIL library of authentic standards) correspond to MSI Level 1; Tier 2 matches (accurate mass and predicted retention time against the Linked Identity library) correspond to MSI Level 2; and Tier 3 matches (accurate mass against MyCompoundID) correspond to putative annotations at MSI Level 2 to 3.

### Statistical analysis

Performance data were analyzed by Welch two sample *t* tests in R version 4.3.3 (28) via RStudio 2026.1.2.418. Initial body weight was used at allocation to assign ewes to the two treatments so that mean starting weight was balanced between groups and allocation bias was minimized, rather than as a formal blocking factor; because initial body weight did not differ between groups (*p* = 0.99), it was not included as a block term in the analysis. LC–MS peak intensities were imported into MetaboAnalyst 6.0 (29) and processed as follows: per-sample median normalization to correct for inter-sample concentration differences, log10 transformation to reduce heteroscedasticity, and auto scaling (mean centering with division by standard deviation of each feature) to render features comparable in magnitude prior to multivariate analysis. Group structure was visualized by partial least squares discriminant analysis (PLS-DA); model fit was reported as *R^2^* (goodness of fit on training data) and *Q^2^* (predictive ability estimated by 5-fold cross-validation), and separation was tested against 100 permutations of class labels using both prediction accuracy and between/within group separation distance as test statistics. Metabolite features contributing most strongly to group structure were ranked by variable importance in projection (VIP) scores with a working threshold of VIP ≥ 1.0. Statistical inference on differential abundance was based on univariate volcano plot analysis with FDR adjustment (see below) rather than on the multivariate PLS-DA model. Differentially abundant metabolites were identified using volcano plot analysis and defined as features with an absolute fold change ≥ 1.5 and a false discovery rate (FDR < 0.05). This combined statistical and effect-size threshold was used to prioritize metabolite features with both biological relevance and statistical support. Pathway enrichment was performed against the *Ovis aries* KEGG library using the global test for over-representation and relative betweenness centrality for topology analysis. Pathway enrichment analysis was considered significant at *p* ≤ 0.10 because it was intended as an exploratory, hypothesis-generating analysis. Given the limited number of significantly altered metabolites that typically map to individual metabolic pathways in untargeted metabolomics, this threshold was selected to reduce the likelihood of overlooking biologically meaningful pathways for subsequent investigation. Metabolic pathways with –log10(P) ≥ 1.0 (equivalent to *p* ≤ 0.10) were considered statistically significant. For biomarker discovery, receiver operating characteristic (ROC) curve analysis was performed on the broader FDR-significant metabolite pool (FDR < 0.05, regardless of the |fold change| ≥ 1.5 magnitude threshold used to define differential abundance), with area under the curve (AUC) > 0.85 selected as the threshold for individual candidate biomarkers; combined biomarker panel performance was evaluated using the random forest classification algorithm implemented within MetaboAnalyst (29). Individual ROC curves and AUC values were computed with the pROC package 1.18.5 in R.

Spearman rank correlations between log10 transformed candidate biomarker abundance and ADG or feed:gain were used because of the small sample size (*n* = 18) and non-normal distribution of metabolite intensities, and were generated using the Hmisc 5.1-3 (30), ggplot2 3.5.2 (31), and ggpubr 0.6.0 (32) packages in R. Multiple linear regression models (lm function) were fit to test whether MEO supplementation status and the four candidate biomarkers were independently associated with ADG and feed:gain; variance inflation factors (VIF) were calculated using the car package 3.1-2 (33) to assess multicollinearity. Given the small sample size (*n* = 18) and the high observation-to-predictor ratio sensitivity of multiple regression, model R-squared values should be interpreted as descriptive of within-sample fit rather than out-of-sample predictive performance, and cross-validation in larger cohorts is required to confirm biomarker generalizability. The sample size of *n* = 9 per group was consistent with published discovery-scale plasma metabolomics studies in sheep (18, 19) and represents an exploratory cohort for candidate biomarker identification rather than a confirmatory trial.

## Results

The growth performance data revealed that dietary supplementation of MEO influenced final body weight (kg), total weight gain (kg), average daily gain (kg/day), and feed:gain of ewes (*p* < 0.05). The initial body weight (kg) did not differ significantly (*p* = 0.99), reflecting a comparable baseline weight between CON and MEO ewes. Daily feed offered did not differ significantly between groups (*p* = 0.99), indicating that differences in feed conversion efficiency between treatments reflected altered nutrient utilization rather than differential intake. However, MEO significantly increased the final body weight of ewes (*p* = 0.047) (Table 1). In addition, total weight gain of ewes was improved by MEO supplementation (*p* < 0.01) (Table 1). Similarly, MEO supplementation increased the average daily gain of ewes (*p* < 0.01) (Table 1). Furthermore, the feed efficiency of ewes was enhanced by MEO supplementation. This is attributed to the reduced feed:gain exhibited by MEO ewes compared with the CON group (*p* = 0.01) (Table 1). Overall, this performance result indicates that supplementation of ewes with MEO has a positive influence on growth characteristics and efficiency in nutrient use when compared with the control ewes.

**Table 1:** Growth performance of Katahdin ewes fed a basal diet (CON) or the basal diet supplemented with a microfused essential oil blend (MEO) over a 35-d feeding period.

Variables	CON	MEO	SE	*p*-value
Initial body weight (kg)	26.32	26.35	1.80	0.99
Final body weight (kg)	33.73^b^	37.23^a^	1.61	0.05
Total weight gain (kg)	7.41^b^	10.88^a^	0.32	<0.01
ADG (kg/day)	0.21^b^	0.31^a^	0.01	<0.01
Daily feed offered (kg/day)	1.18	1.19	0.08	0.99
Feed:gain	5.74^a^	3.82^b^	0.54	0.01

Within a row, means without a common superscript differ (*p* < 0.05). CON, ewes fed the basal diet; MEO, ewes fed the basal diet supplemented with a microfused essential oil blend; ADG, average daily gain; SE, standard error of the mean. 95% confidence intervals for the MEO minus CON difference (Welch two sample t, *n* = 9 per group): final body weight 0.05 to 6.95 kg; total weight gain 2.77 to 4.17 kg; average daily gain 0.08 to 0.12 kg/day; initial body weight −3.85 to 3.91 kg.

### Plasma metabolomic profile

**Figure 1:**
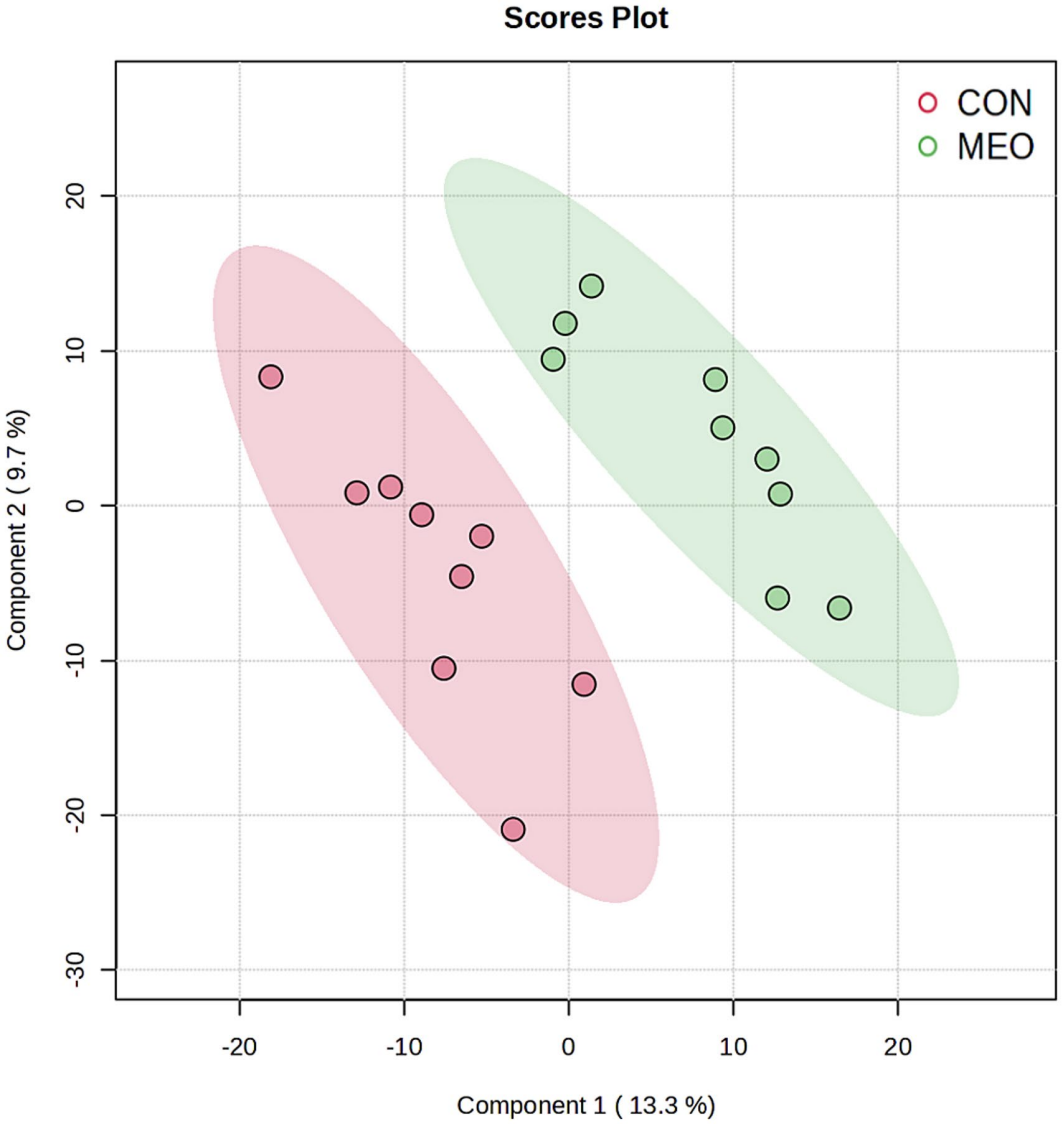
Partial least squares discriminant analysis (PLS-DA) scores plot of the plasma metabolome of CON and MEO ewes (*n* = 9 per group). Components 1 and 2 explained 13 and 10% of the variance in the peak intensity matrix. Model cross-validation performance was limited (*R*2 = 0.92, *Q*2 = −0.24 for two components; permutation *p* = 1.0 at 100 permutations); the plot is therefore presented as a descriptive visualization of group structure rather than as a validated classifier. CON, ewes fed the basal diet (red); MEO, ewes fed the basal diet supplemented with a microfused essential oil blend (green).

**Figure 2:**
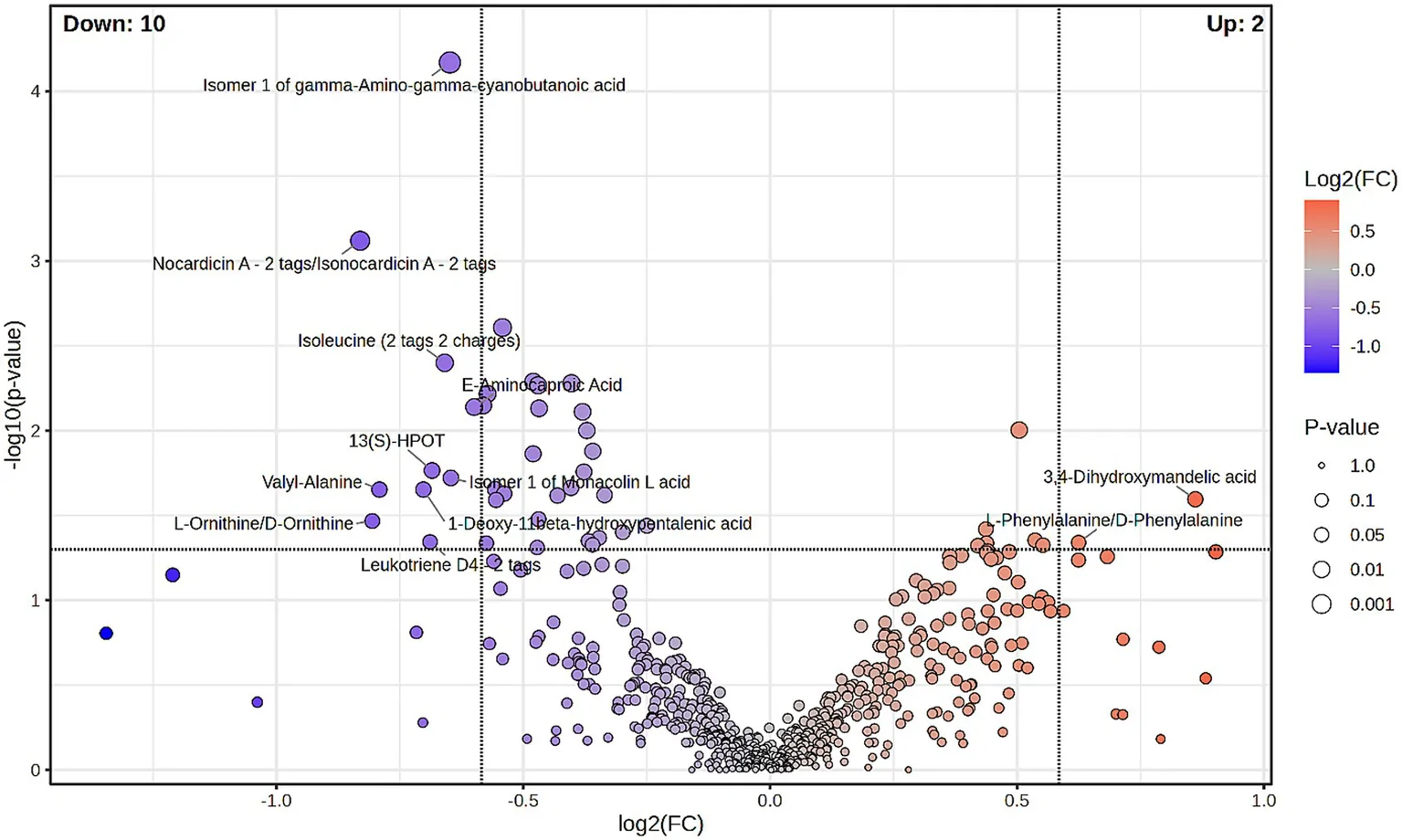
Volcano plot of plasma metabolite differential abundance between CON and MEO ewes (*n* = 9 per group). Each point represents one metabolite plotted as log2 fold change (MEO/CON) against −log10 (*P*). Red points indicate metabolites significantly elevated and blue points indicate metabolites significantly reduced in MEO supplemented ewes (*FDR* <0.05, absolute fold change ≥1.5).

**Figure 3:**
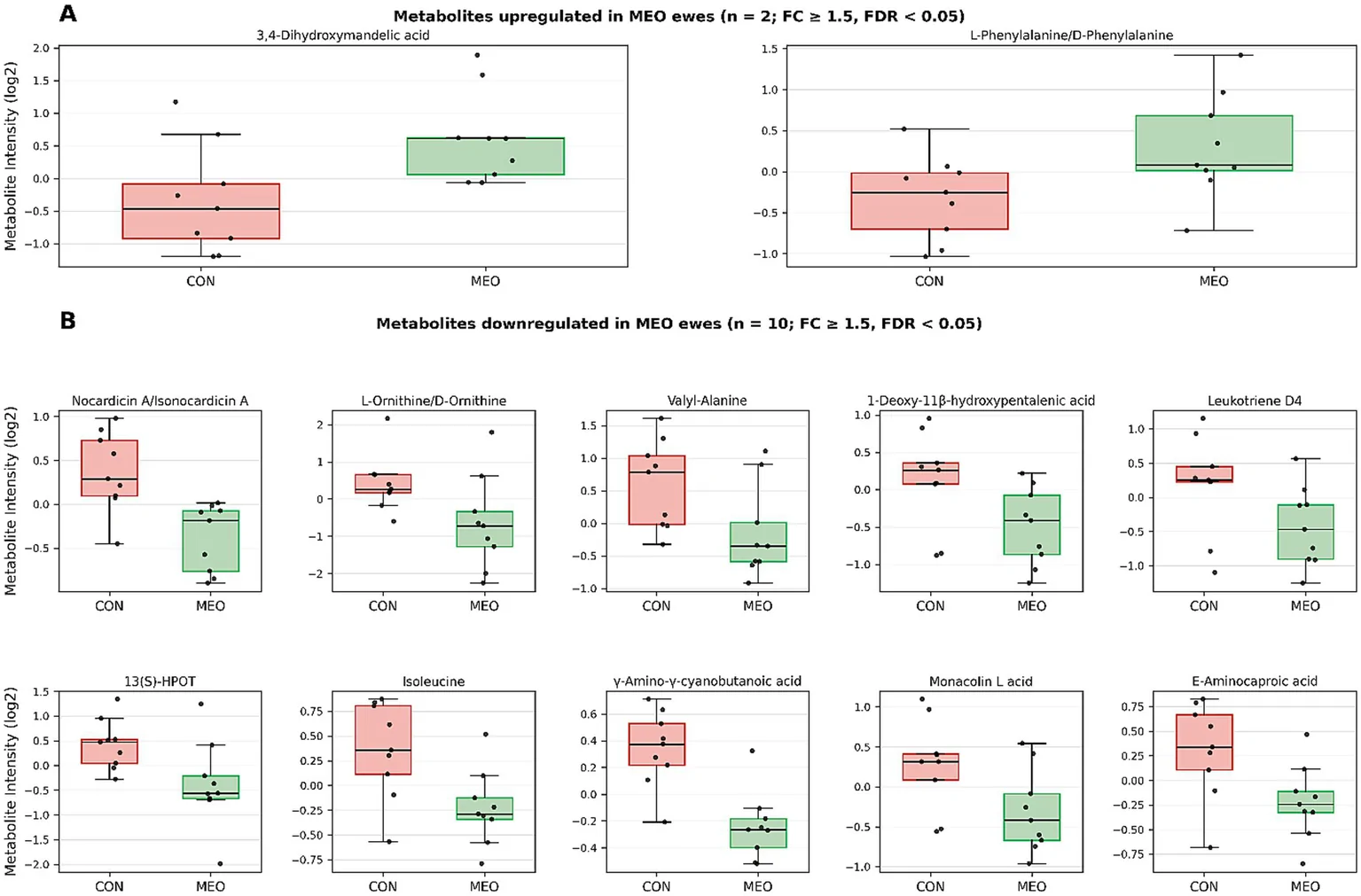
Top differentially abundant plasma metabolites between CON and MEO ewes (*n* = 9 per group). **(A)** Two metabolites (log2) elevated by MEO supplementation. **(B)** Ten metabolites (log2) reduced by MEO supplementation. CON: Ewes fed the basal diet (red); MEO: Ewes fed the basal diet supplemented with a microfused essential oil blend (green).

**Figure 4:**
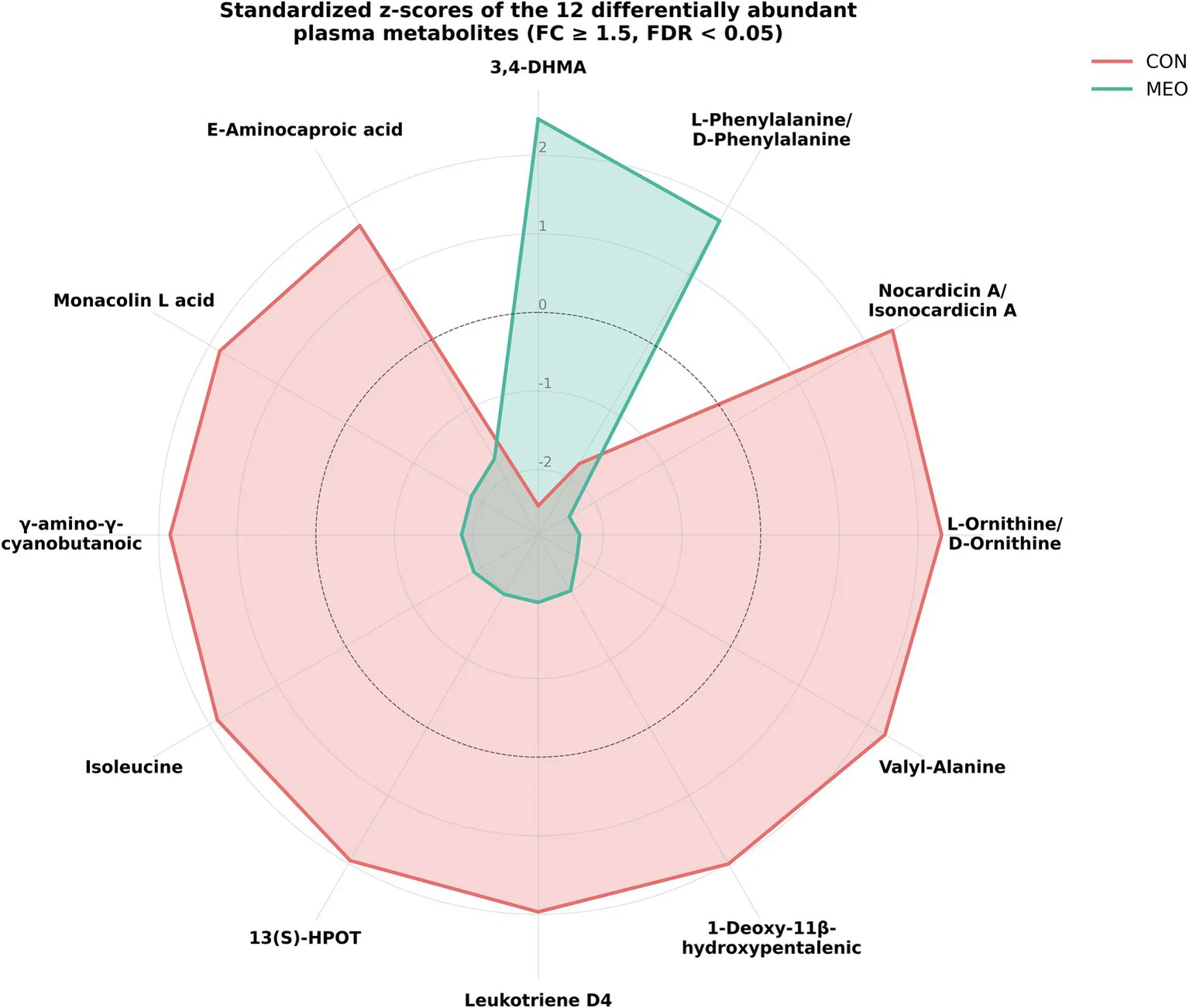
Radar plot of the 12 plasma metabolites differentially abundant between CON and MEO Katahdin ewes (*n* = 9 per group; |*FC*| ≥ 1.5, *FDR* < 0.05). Standardized *z*-scores were computed from log2-transformed group fold changes such that the CON (coral) and MEO (teal) polygons mirror each other around the zero line (dashed). Two metabolites are elevated in MEO ewes (3,4-dihydroxymandelic acid and L-phenylalanine/D-phenylalanine, top of plot), while the remaining 10 metabolites are reduced in MEO ewes. Radial scale in standard-deviation units.

Our LC–MS identified 1,005 known metabolites (Supplementary Table 1) and 601 metabolites remained after filtering. The PLS-DA scores plot showed visible group structure between CON and MEO ewes on the first two components, which explained 13 and 10% of variance in the peak intensity matrix (Figure 1). Because of the small discovery cohort size (*n* = 18) and the high feature dimensionality typical of untargeted plasma metabolomics, model cross-validation performance was limited (*R^2^* = 0.92, *Q^2^* = −0.24 for two components; 5-fold CV accuracy = 0.62; permutation *p* = 1.0 with 100 permutations under both prediction accuracy and between/within group separation distance). The PLS-DA plot is therefore presented as a descriptive visualization of group structure rather than as a validated multivariate classifier, and all statistical claims of differential abundance rely on univariate FDR adjusted testing described below. Furthermore, we identified 12 differentially abundant metabolites (FC ≥ 1.5 and FDR < 0.05) between CON and MEO ewes (Figure 2). A total of 2 metabolites were elevated in MEO ewes (3,4-dihydroxymandelic acid and L-phenylalanine/D-phenylalanine) with fold changes ranging from 1.34 to 1.82 relative to CON. Conversely, 10 metabolites were reduced in MEO ewes including Nocardicin A/Isonocardicin A, L-ornithine/D-ornithine, valyl-alanine, isoleucine, 13(S)-HPOT, leukotriene D4, ε-aminocaproic acid, and *γ*-amino-*γ*-cyanobutanoic acid with fold changes ranging from 0.57 to 0.77 relative to CON (Figure 2; Table 2). The relative abundance of the twelve differential metabolites is presented in Figures 3A,B. Furthermore, the radar visualization (Figure 4) shows the top Z-scored discriminating features between CON and MEO ewes, including 3,4-dihydroxymandelic acid on the MEO side and isomer 1 of *γ*-amino-*γ*-cyanobutanoic acid, valyl-alanine, and Nocardicin A/Isonocardicin A on the CON side.

**Table 2:** Fold change and FDR for differentially abundant plasma metabolites in ewes supplemented with a microfused essential oil blend (MEO) relative to control (CON).

Metabolites	FC (MEO/CON)	FDR
3,4-Dihydroxymandelic acid	1.82	0.025
L-Phenylalanine/D-Phenylalanine	1.54	0.046
ε-aminocaproic acid	0.66	0.007
Isomer 1 of Monacolin L acid	0.64	0.019
Isomer 1 of *γ*-amino-*γ*-cyanobutanoic acid	0.64	<0.001
Isoleucine (2 tags 2 charges)	0.63	0.004
13(S)-HPOT	0.62	0.017
Leukotriene D4 (2 tags)	0.62	0.045
1-deoxy-11*β*-hydroxypentalenic acid	0.61	0.022
Valyl-Alanine	0.58	0.022
L-Ornithine/D-Ornithine	0.57	0.034
Nocardicin A/Isonocardicin A (2 tags)	0.56	<0.001

FC, fold change relative to CON (values >1 indicate greater abundance in MEO ewes; values <1 indicate greater abundance in CON ewes). CON, ewes fed the basal diet; MEO, ewes fed the basal diet supplemented with a microfused essential oil blend.

### Biomarkers and metabolic pathway enrichment

**Figure 5:**
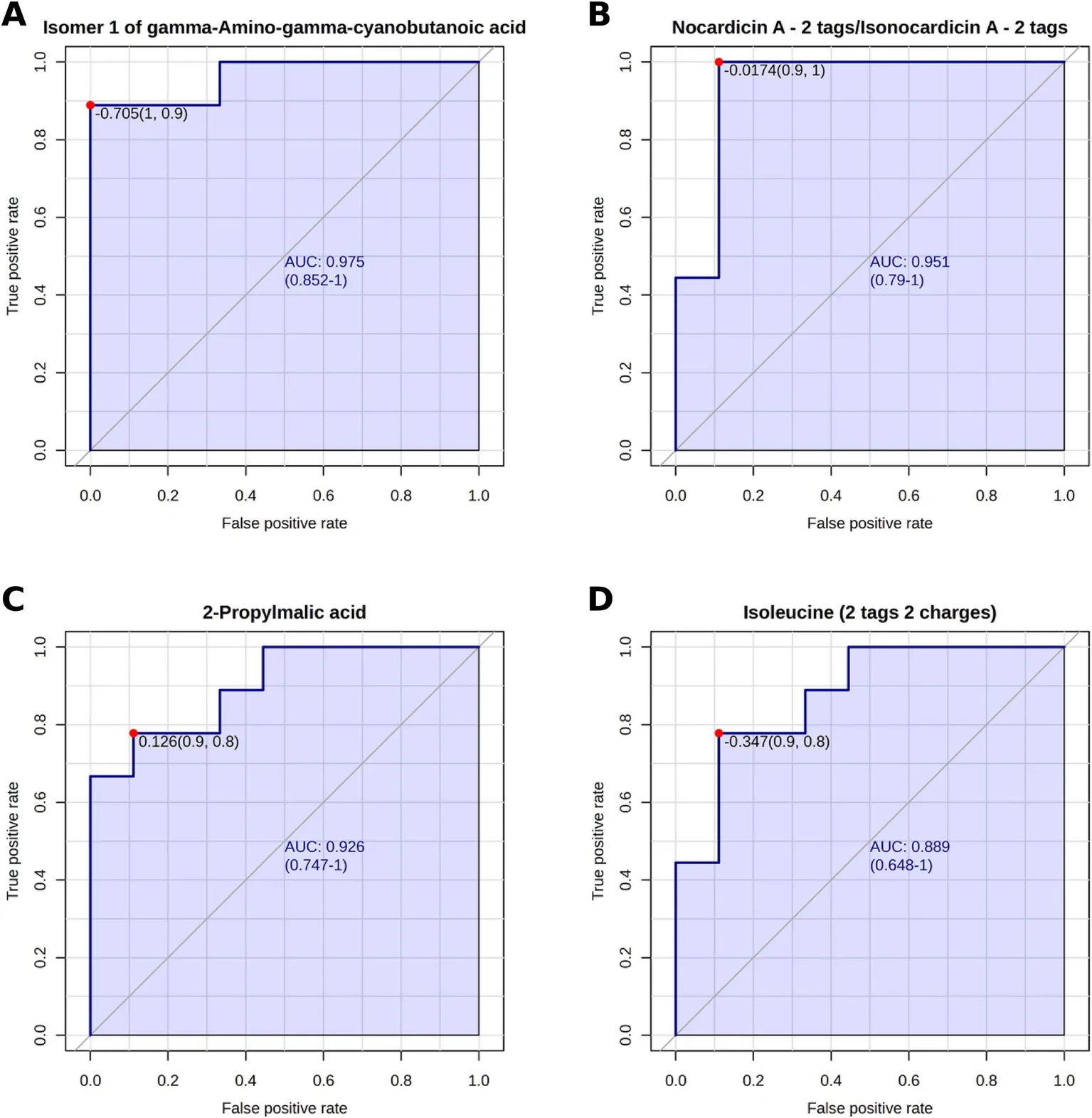
Individual receiver operating characteristic (ROC) curves for the four candidate plasma biomarkers of microfused essential oil blend supplementation in ewes (*n* = 9 CON, *n* = 9 MEO): **(A)** Isomer 1 of *γ*-amino-*γ*-cyanobutanoic acid; **(B)** Nocardicin A/Isonocardicin A; **(C)** 2-Propylmalic acid; and **(D)** Isoleucine.

**Figure 6:**
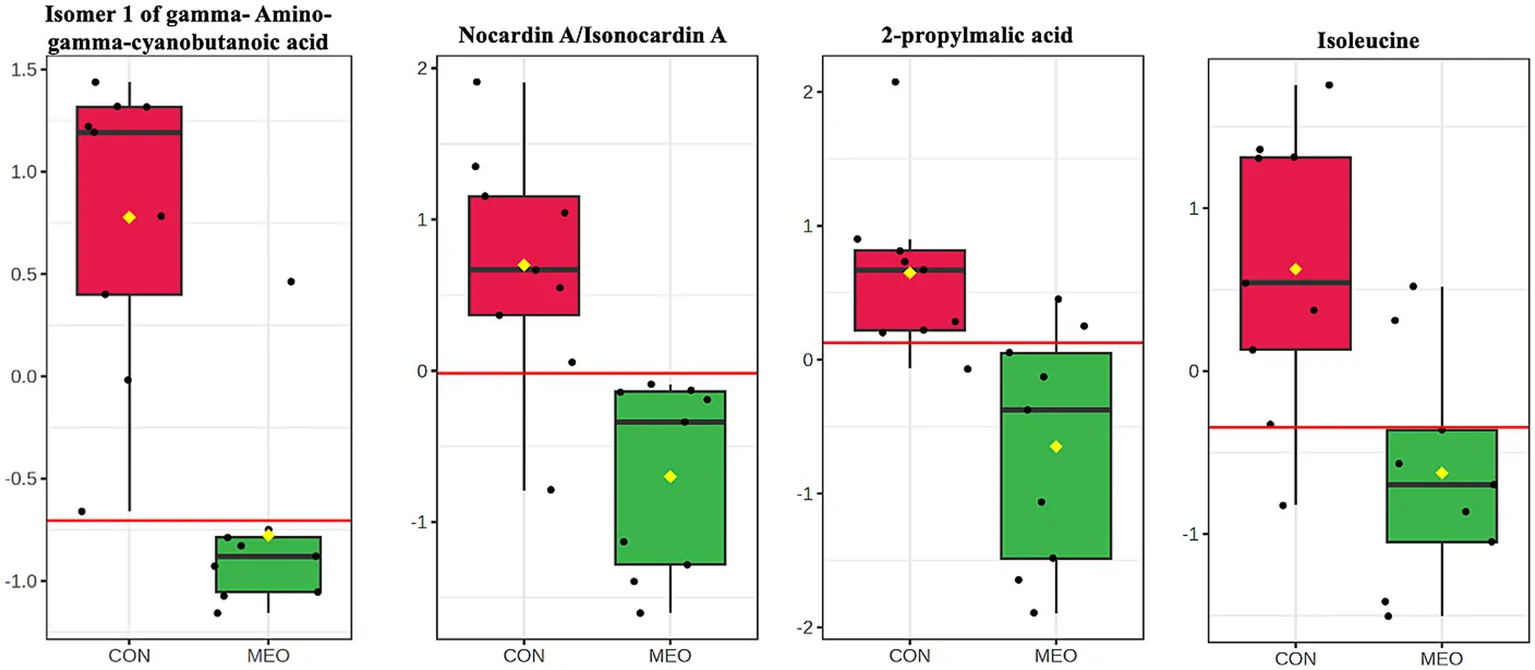
Relative distribution of the four candidate plasma biomarkers of MEO supplementation in ewes (*n* = 9 per group). Box plots show the median, interquartile range, and minimum to maximum values of median normalized log10 transformed peak intensities. CON, ewes fed the basal diet; MEO, ewes fed the basal diet supplemented with a microfused essential oil blend.

**Figure 7:**
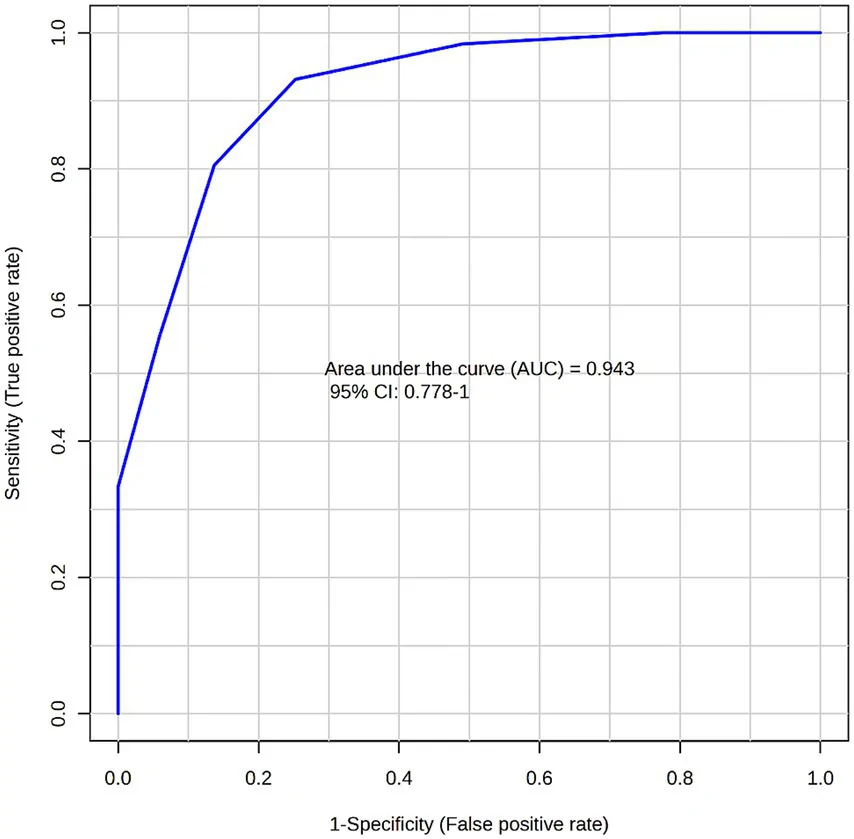
Combined four biomarker panel receiver operating characteristic (ROC) curve generated by random forest classification in MetaboAnalyst 6.0 (*n* = 9 CON, *n* = 9 MEO). The combined panel achieved an area under the curve (AUC) of 0.943, indicating strong discriminatory capacity between MEO supplemented and control ewes.

**Figure 8:**
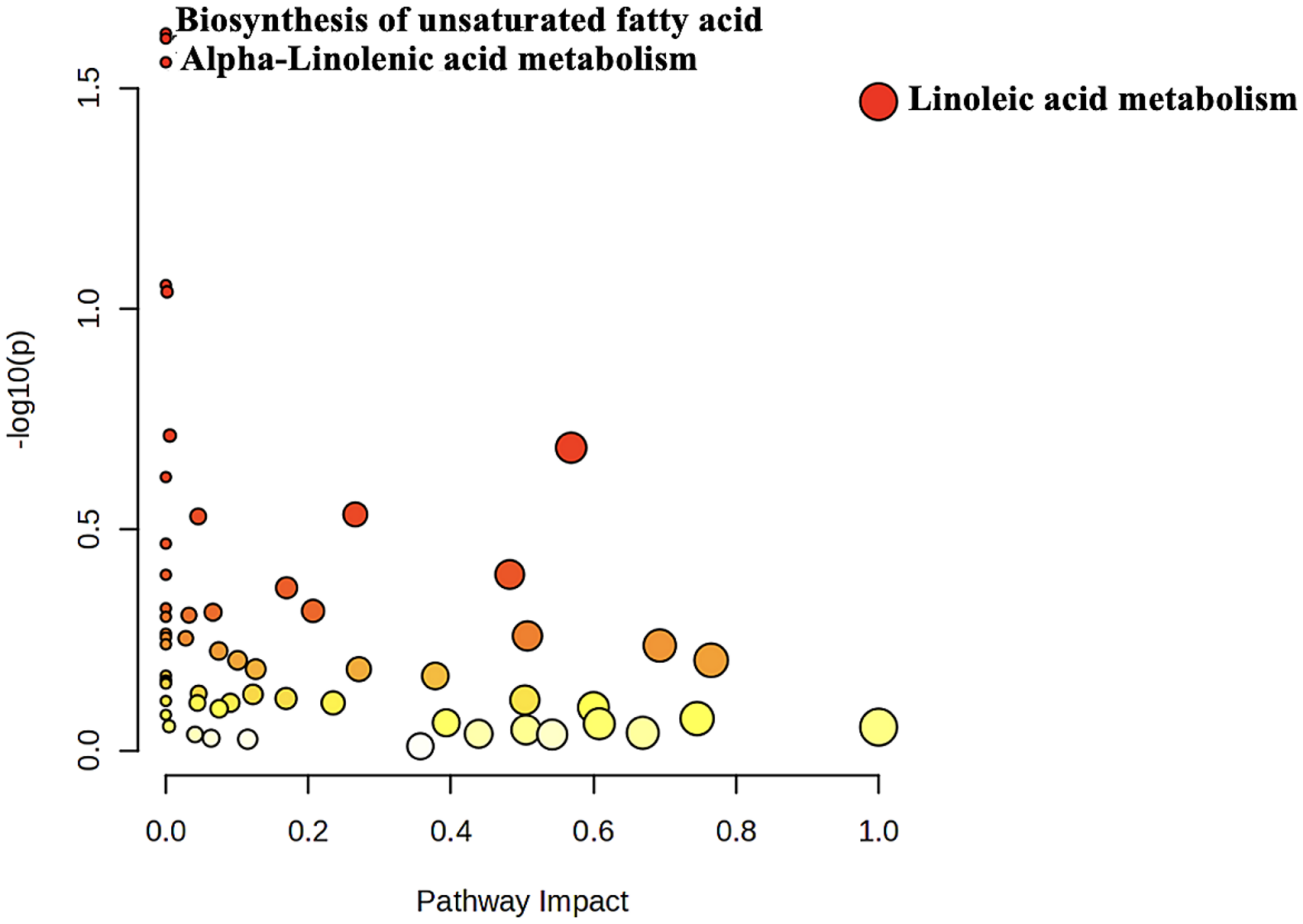
Differential metabolic pathway enrichment between plasma metabolomes of CON and MEO ewes generated by the MetaboAnalyst 6.0 pathway module against the KEGG *Ovis aries* library. Larger and warmer colored nodes indicate pathways with greater biological effect. Three polyunsaturated fatty acid metabolism pathways were significantly enriched in MEO ewes: Linoleic acid metabolism, α-linolenic acid metabolism, and biosynthesis of unsaturated fatty acids (Metabolic pathways with –log10(*P*) ≥ 1.0 (equivalent to *p* ≤ 0.10) were considered statistically significant).

Our biomarker discovery analysis identified four candidate metabolites associated with MEO supplementation drawn from the broader FDR-significant metabolite pool (FDR < 0.05; AUC ≥ 0.85), including *γ*-amino-*γ*-cyanobutanoic acid, Nocardicin A/Isonocardicin A, 2-propylmalic acid, and isoleucine. The associated AUC values reflect sufficient specificity and sensitivity to discriminate between CON and MEO ewes (Figure 5), and all four markers were lower in MEO supplemented ewes, a pattern consistent with altered aromatic amino acid turnover and redox metabolism (Figure 6). When combined into a single classifier, the four-metabolite panel achieved an AUC of 0.943 (95% CI 0.778 to 1.000), consistent with the strong individual biomarker AUCs and the significant univariate FDR-adjusted differential abundance patterns (Figure 7). Pathway enrichment analysis identified three coordinately enriched polyunsaturated fatty acid pathways in MEO ewes: biosynthesis of unsaturated fatty acids, α-linolenic acid metabolism and linoleic acid metabolism (Metabolic pathways with –log10(P) ≥ 1.0 (equivalent to *p* ≤ 0.10); Figure 8). Together, these three pathways reflect a remodeling of the plasma polyunsaturated fatty acid pool in MEO ewes, spanning both the omega-6 (linoleic acid) and omega-3 (α-linolenic acid) branches, and are directly supported by the concurrent depletion of leukotriene D4 and 13(S)-HPOT observed in the differential metabolome.

### Association of biomarkers and ewes’ performance traits

To assess the biological importance of candidate biomarkers, we performed spearman correlation analysis between metabolite abundance (log transformed) and average daily gain (kg/day) and feed efficiency (feed:gain).

**Figure 9:**
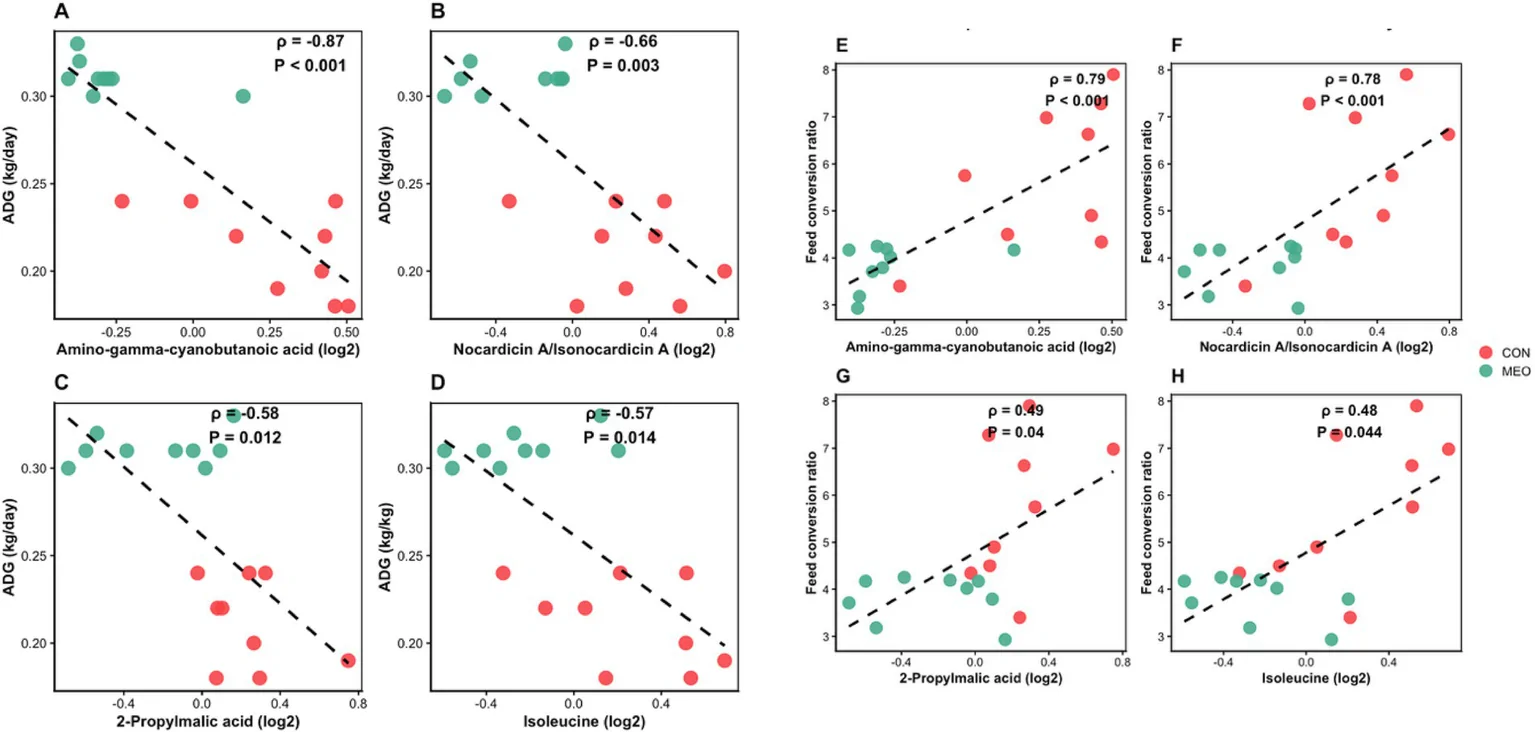
Spearman rank correlations between the four candidate plasma biomarkers and growth performance traits in ewes (*n* = 9 CON, *n* = 9 MEO). Panels A to D show negative correlations with average daily gain (ADG, kg/day): **(A)** *γ*-amino-*γ*-cyanobutanoic acid; **(B)** Nocardicin A/Isonocardicin A; **(C)** 2-Propylmalic acid; **(D)** Isoleucine. **(E–H)** Show positive correlations with feed:gain: **(E)** *γ*-amino-*γ*-cyanobutanoic acid; **(F)** Nocardicin A/Isonocardicin A; **(G)** 2-Propylmalic acid; **(H)** Isoleucine. Each point represents one ewe; the dashed line shows the linear regression fit.

A negative relationship was observed between ADG and *γ*-amino-*γ*-cyanobutanoic acid (*ρ* = −0.87, *p* < 0.001), Nocardicin A/Isonocardicin A (*ρ* = −0.66, *p* < 0.001), 2-propylmalic acid (*ρ* = −0.58, *p* = 0.01), and isoleucine (*ρ* = −0.57, *p* = 0.01) (Figures 9A–D). Ewes with lower concentrations of these metabolites have higher ADG (kg/day). In contrast, positive associations with feed:gain were observed for *γ*-amino-*γ*-cyanobutanoic acid (*ρ* = 0.79, *p* < 0.001), Nocardicin A/Isonocardicin A (*ρ* = 0.78, *p* < 0.001), 2-propylmalic acid (ρ = 0.49, *p* = 0.04), and isoleucine (*ρ* = 0.48, *p* = 0.04) (Figures 9E–H). To quantify the independent contribution of each biomarker to variation in ADG and feed efficiency, multiple linear regression models were fitted with MEO supplementation status and the four candidate biomarkers as predictors. Our results showed that variance inflation factors (VIF = 3.46 to 6.25) did not reflect severe multicollinearity among the candidate biomarkers. Nevertheless, the model explained about 95% of variation in ADG (*R^2^* = 0.95, adjusted *R^2^* = 0.93, *p* < 0.01). In addition, significant predictors include MEO supplementation (*β* = 0.06; *p* < 0.01), *γ*-amino-*γ*-cyanobutanoic acid (*β* = −0.07; *p* < 0.01), and isoleucine (*β* = −0.06; *p* = 0.02) while after accounting for other metabolites, Nocardicin A/Isonocardicin A (*β* = 0.02; *p* = 0.15) and 2-propylmalic acid (*β* = 0.04; *p* = 0.12) were not independently associated with ADG, but may be involved in overlapping biological metabolic pathways. A similar pattern was observed for feed efficiency, with 78% of variance explained (*R^2^* = 0.78, adjusted *R^2^* = 0.68, *p* < 0.01) except that MEO supplementation was not significant (*β* = 0.48; *p* = 0.55). This may suggest that variation in ewe’s feed efficiency is accounted for by these candidate biomarkers. Furthermore, *γ*-amino-*γ*-cyanobutanoic acid (*β* = 3.48; *p* = 0.01) and isoleucine (*β* = 3.53; *p* = 0.02) predicted feed efficiency, whereas Nocardicin A/Isonocardicin A (*β* = −0.12; *p* = 0.90) and 2-propylmalic acid (*β* = −2.51; *p* = 0.08) were not independently associated with feed efficiency (Tables 3, 4). Taken together, these results revealed that *γ*-amino-*γ*-cyanobutanoic acid and isoleucine have strong associations with the MEO ewe phenotype.

**Table 3:** Multiple linear regression of average daily gain (kg/day) and feed:gain on microfused essential oil blend (MEO) supplementation status and the four candidate plasma biomarkers in ewes (*n* = 18).

Predictors	ADG (kg/day)		Feed:gain	
	*β*	*P-*value	*β*	*P-*value
Intercept	0.23	<0.01	4.56	<0.01
MEO supplementation	0.06	<0.01	0.48	0.55
*γ*-amino-*γ*-cyanobutanoic acid	−0.07	0.00	3.48	0.01
Nocardicin A/Isonocardicin	0.02	0.15	−0.12	0.90
2-Propylmalic acid	0.04	0.12	−2.51	0.08
Isoleucine	−0.06	0.02	3.53	0.02

*β* is the multiple linear regression coefficient for each predictor. ADG, average daily gain (kg/day). Significant predictor coefficients (*P* < 0.05) are shown in bold.

**Table 4:** Model fit statistics for the multiple linear regression models of average daily gain and feed:gain in ewes (*n* = 18).

Metrics	ADG (kg/day) model	Feed:gain model
*R* ^2^	0.95	0.78
Adjusted *R*^2^	0.93	0.68
*P*-value	<0.01	<0.01
Residual standard error	0.01	0.83

*R*^2^ is the proportion of variance in the outcome explained by the model. Adjusted *R*^2^ adjusts for the number of predictors.

## Discussion

This study profiled the plasma metabolome of Katahdin ewes supplemented with a microfused essential oil blend (MEO) and integrated the metabolite signature with growth performance to identify candidate biomarkers of supplementation status. MEO supplementation produced concurrent improvements in growth phenotype and a coordinated remodeling of the plasma metabolome, comprising 12 differentially abundant metabolite features, four high-ranking candidate biomarkers (combined AUC =  0.943), and pathway enrichment converging on three polyunsaturated fatty acid pathways (linoleic acid metabolism, α-linolenic acid metabolism, and biosynthesis of unsaturated fatty acids). The resulting plasma signature places phytogenic essential oil action within an interpretable systemic metabolic framework and identifies metabolite biomarkers independently associated with average daily gain and feed efficiency in MEO supplemented ewes.

The higher final body weight, total weight gain, ADG, and lower feed:gain observed in MEO ewes are consistent with previous reports that essential oil and related phytogenic additives improve nutrient utilization, feed efficiency, and growth performance in ruminants (3, 13, 34, 35). The performance advantage likely arises from convergent mechanisms acting on host energy and nitrogen handling. Essential oil bioactives enhance the activity of intestinal digestive enzymes, support mucosal integrity, and increase systemic antioxidant capacity (8, 14), and at the dose used in the present study, comparable EO supplementation in sheep has been associated with improved nutrient digestibility and altered systemic metabolite profiles (8). Microfusion of the bioactive matrix is expected to reinforce these effects by protecting volatile constituents from gastrointestinal degradation and extending their effective dose window (22).

Notably, MEO supplementation increased average daily gain by approximately 48% relative to the control while daily feed offered did not differ between groups, indicating that the response reflects improved feed efficiency, that is, a lower feed:gain, rather than greater intake. Such an effect at constant intake is biologically plausible through the mechanisms described above, namely enhanced digestive enzyme activity, improved mucosal integrity and nutrient absorption, and increased antioxidant capacity, which together reduce nutrient and energy losses and partition a larger fraction of absorbed nutrients toward growth. Nevertheless, a change of this magnitude is large relative to typical essential oil responses in ruminants and should be interpreted with caution given the exploratory sample size of nine ewes per group; because rumen fermentation, nutrient digestibility, and feeding behavior were not measured, these mechanisms are presented as hypotheses that require confirmation in larger studies incorporating direct digestibility and fermentation measurements.

A defining feature of the differential plasma metabolome is that 10 of the 12 differentially abundant features were reduced in MEO ewes. The reduction pattern spans several biochemical themes: branched chain amino acid related features (isoleucine, valyl-alanine), a nitrogen and amino acid pool marker (L-ornithine/D-ornithine), a GABA related analog (isomer 1 of *γ*-amino-*γ*-cyanobutanoic acid), linoleic acid derived lipid peroxidation products (13(S)-HPOT, leukotriene D4), and microbial origin secondary metabolites (Nocardicin A/Isonocardicin A, isomer 1 of Monacolin L acid, 1-deoxy-11*β*-hydroxypentalenic acid). Rather than indicating reduced systemic production, we speculate that the depletion observed may reflect enhanced peripheral utilization of the circulating metabolites, changes in microbial metabolism, altered nutrient absorption and liver metabolism, or a proposed combination of these mechanisms. A comparable plasma metabolite depletion pattern has been reported in lambs divergent for feed efficiency, where genetically efficient animals showed lower circulating concentrations of L-threonine, L-serine, L-leucine, and *β*-hydroxyisovalerate (19). A conceptually parallel broad amino acid depletion has also been observed in the plasma of insulin dysregulated horses following 6 weeks of essential oil supplementation, where glycine, serine, threonine metabolism, cysteine and methionine metabolism, and *β*-alanine metabolism were among the most significantly enriched pathways (21), indicating that essential oil driven remodeling of the circulating amino acid pool is not restricted to ruminant systems.

A closer inspection of the reduced metabolite set reveals two coherent themes tied to ruminal and gut microbial biology. The three microbial origin secondary metabolites depleted in MEO ewes span both bacterial and fungal producers: Nocardicin A/Isonocardicin A is a monocyclic *β*-lactam antibiotic produced by Actinobacteria (originally isolated from *Nocardia* and now attributed to *Actinomadura*), 1-deoxy-11*β*-hydroxypentalenic acid is a pentalenolactone family sesquiterpene biosynthetic intermediate produced by *Streptomyces* species, and isomer 1 of Monacolin L acid is a polyketide of the monacolin family produced by *Aspergillus terreus* and *Monascus purpureus* with structural homology to statin family HMG-CoA reductase inhibitors. Carvacrol, thymol, and cinnamaldehyde, the principal phenolic monoterpenes and phenylpropanoids of oregano, thyme, and cinnamon essential oils, have broad-spectrum antimicrobial activity against Gram-positive bacteria including Actinobacteria (4, 7, 9) and against filamentous fungi including *Aspergillus* and *Monascus* species (4, 7), with dietary essential oil supplementation reported to selectively modulate ruminal Gram-positive bacterial populations in beef cattle (10). The concurrent depletion of these putative microbial-derived molecules may be consistent with essential oil’s mediated suppression of microbial activity and dynamics after MEO supplementation. In parallel, ε-aminocaproic acid (6-aminohexanoic acid), a lysine analog whose most likely biological source in ruminant plasma is turnover of epsilon-poly-L-lysine, a cationic antimicrobial polypeptide produced by some *Streptomyces* species and hydrolyzed to lysine and ε-aminocaproic acid-like fragments by bacterial and host peptidases (36), was reduced in MEO ewes (FC = 0.66, FDR = 0.007). Ruminal small amines and short-chain organic acids of microbial origin are known to cross the ruminal epithelium via cation-selective transporters and enter the portal circulation (37, 38), where they contribute to peripheral plasma composition after partial hepatic first-pass metabolism, providing the biological pathway linking ruminal microbial catabolic activity to plasma metabolite appearance. Reduced plasma ε-aminocaproic acid in MEO ewes could indicate reduced ruminal turnover, which could mirror the concurrent depletion of the *Streptomyces*-derived pentalenolactone intermediate 1-deoxy-11*β*-hydroxypentalenic acid, and could raise the possibility of reduced ruminal microbial proteolytic activity following essential oil supplementation. Formal confirmation would require paired rumen fluid and plasma metabolomics with absolute quantification of ε-aminocaproic acid across ruminal, portal, and peripheral compartments. Together, these observations converge on essential oil driven modulation of the ruminal microbial community, with concurrent depletion of microbial secondary metabolites and reduced microbial proteolysis, and provide a mechanistic complement to the amino acid and polyunsaturated fatty acid pathway signals developed above.

The 2 metabolite features upregulated in MEO ewes both point to altered aromatic amino acid and catecholamine metabolism. The upregulation of D/L-phenylalanine, an aromatic amino acid precursor of protein and catecholamine synthesis, together with 3,4-dihydroxymandelic acid (DOMA), a well-characterized catabolite of dopamine and norepinephrine and a downstream product of monoamine oxidase and catechol-O-methyltransferase activity, indicates altered systemic catecholamine turnover in MEO ewes. This concurrent elevation may be consistent with phytogenic modulation of the phenylalanine to tyrosine to catecholamine biosynthesis axis together with accelerated catecholamine degradation, an axis with established links to gut motility, sympathetic tone, immune competence, and stress physiology in ruminants. Modulation of aromatic amino acid handling has previously been reported in oregano essential oil supplemented sheep with parallel improvements in intestinal barrier function and reduced inflammation (8). Together with the depletion of branched chain amino acid related features (isoleucine, valyl-alanine) and putative microbial derived metabolites, this profile may suggest that systemic aromatic amino acid and catecholamine changes could be a putative mechanism through which MEO supplementation may influence the physiology and performance of growing ewes.

Pathway analysis identified three coordinately enriched polyunsaturated fatty acid (PUFA) metabolism pathways in MEO ewes: linoleic acid metabolism (*p* = 0.034, pathway impact = 1), α-linolenic acid metabolism (*p* = 0.028), and biosynthesis of unsaturated fatty acids (*p* = 0.024; Figure 8). Together, these three pathways point to a systemic remodeling of the plasma PUFA pool that spans both the omega-6 (linoleic acid) and omega-3 (α-linolenic acid) branches (39, 40). Direct metabolite evidence for this remodeling is present in the differential metabolome: leukotriene D4, a pro inflammatory lipid mediator arising from arachidonic acid downstream of linoleic acid, was reduced in MEO ewes (FC = 0.62, FDR = 0.045), together with the linoleic acid derived peroxidation product 13(S)-HPOT (FC = 0.62, FDR = 0.017). n-3 and n-6 PUFA derivatives regulate inflammation, membrane biogenesis, cellular signaling, and mitochondrial energy efficiency (40–42), and their coordinated plasma depletion in MEO ewes may indicate enhanced peripheral incorporation into tissue phospholipid pools and reduced downstream eicosanoid production, consistent with the reduced inflammatory tone reported in essential oil supplemented sheep (8). However, because inflammatory cytokines, oxidative stress biomarkers, and antioxidant enzyme activities were not measured in the present study, this interpretation should be regarded as a biologically plausible hypothesis rather than a demonstrated effect. Independent identification of α-linolenic acid metabolism as a main enriched plasma pathway in probiotic supplemented fattening sheep achieving superior average daily gain (43) further supports the present result as a candidate metabolomic signature of feed additive driven metabolic improvement in sheep. Remarkably, the same three pathway PUFA signature, comprising biosynthesis of unsaturated fatty acids, α-linolenic acid metabolism, and linoleic acid metabolism with pathway impact = 1, was independently identified by chemical isotope labeling LC MS in the plasma of insulin dysregulated horses receiving essential oil supplementation, where a concurrent decrease in circulating linoleic acid also accompanied improved insulin sensitivity (21). This cross-species convergence on identical PUFA metabolism pathways using the same analytical platform suggests that the observed PUFA signature may represent a conserved plasma metabolic response to essential oil supplementation across ruminant and monogastric species.

Receiver operating characteristic analysis identified four plasma metabolites as candidate biomarkers of MEO supplementation. The discriminatory power and panel composition are consistent with prior plasma and serum metabolomics in sheep that have used PLS-DA and ROC AUC frameworks to nominate candidate biomarkers of residual feed intake and carcass merit (18, 19). All four candidate biomarkers were lower in MEO ewes and span branched chain, *γ* amino, aromatic, and secondary metabolite pathways. Reduced 2-propylmalic acid may indicate alterations in systemic branched-chain amino acid metabolism, potentially reflecting changes in intermediary metabolic pathways (42, 44). Similarly, the lower abundance of Nocardicin A/Isonocardicin A in MEO-fed ewes could signify altered circulating levels of microbial-derived or xenobiotic-associated secondary metabolites. This observation is consistent with the proposed enhanced delivery and stability of bioactive compounds afforded by microfused encapsulation (22). However, because this metabolite was identified through untargeted metabolomics and the rumen microbiome was not characterized, its biological source and mechanistic importance need clarification. Although the functional role of *γ*-amino-*γ*-cyanobutanoic acid in ruminants remains poorly characterized, its strong single biomarker discriminatory power and its strongest correlation with ADG (*ρ* = −0.87, *p* < 0.001) and feed:gain (*ρ* = 0.79, *p* < 0.001) identify it as a priority target for future targeted metabolomics validation.

All four candidate biomarkers were negatively correlated with ADG and positively correlated with feed:gain (Figure 9). Because lower feed:gain reflects greater feed efficiency, opposite sign correlations between the biomarkers and ADG versus feed:gain are biologically consistent and indicate that lower plasma concentrations of these metabolites may be associated with superior growth and efficiency. *γ*-amino-*γ*-cyanobutanoic acid showed the strongest correlation with ADG (*ρ* = −0.87, *p* < 0.001), followed by Nocardicin A/Isonocardicin A, 2-propylmalic acid, and isoleucine. Multiple regression analysis controlling for treatment effect identified *γ*-amino-*γ*-cyanobutanoic acid and isoleucine as independent predictors of both ADG and feed efficiency (95% of ADG variation and 78% of feed:gain variation explained). Biologically, a unit increase in plasma *γ*-amino-*γ*-cyanobutanoic acid or isoleucine was associated with an estimated 0.07 kg/day or 0.06 kg/day reduction in ADG and an estimated 3.48 or 3.53 unit increase in feed:gain, respectively. Combined with low multicollinearity (VIF 3.46 to 6.25), these results indicate that the two metabolites capture distinct metabolic information about MEO associated growth performance in ewes.

Comparable metabolic signatures have been documented across ruminant and monogastric livestock species supplemented with essential oils and related phytogenic additives. In beef cattle, combined essential oil and alpha-amylase supplementation improved growth performance and carcass characteristics with concurrent shifts in ruminal fermentation profile (45), while microencapsulated essential oil blends produced consistent effects on intake and methane emissions in progressively adapted sheep (34). In dairy cows, a microencapsulated blend of essential oils and capsaicin increased milk yield, altered ruminal fatty acid biohydrogenation, and modified systemic energy metabolism (35). Parallel work in dairy cows further reported coordinated shifts in ruminal fatty acid metabolism and milk fatty acid profile following phytochemical supplementation, with enrichment of conjugated linoleic acid production (46). A conceptually parallel broad amino acid depletion and PUFA remodeling pattern has also been observed in the plasma of insulin dysregulated horses following 6 weeks of essential oil supplementation (21), indicating that phytogenic driven remodeling of the circulating metabolite pool is not restricted to ruminant systems. These convergent findings across species and analytical platforms support the plasma metabolomic signature identified in the present MEO ewes as a reproducible metabolomic footprint of essential oil action in livestock.

Several limitations should be considered when interpreting these findings. Although over 8,600 metabolite peak pairs were initially detected, only 12 metabolites remained significant after FDR correction and fold-change filtering, indicating that MEO supplementation elicited relatively modest systemic metabolic alterations rather than a broad shift of the plasma metabolome, consistent with the subtle systemic effects expected from a nutritional intervention. A significance threshold of *p* ≤ 0.10 was used for pathway enrichment because the analysis was exploratory and intended to generate biological hypotheses rather than establish definitive mechanistic conclusions; although this more liberal threshold increases the possibility of false-positive pathway enrichment, it reduces the likelihood of overlooking biologically relevant pathways in untargeted metabolomics datasets. Compositing precluded assessment of temporal metabolic responses, although it presents a robust overall representation of the cumulative systemic metabolic adaptation associated with MEO supplementation. Given our sample size (*N* = 18), the ROC-derived AUC values should be interpreted as exploratory measures of putative biomarkers rather than definitive estimates of diagnostic performance. Although the regression models explained a large proportion of the variation in growth performance, the relatively small sample size compared with the number of predictors increases the likelihood of model overfitting, so these associations should be regarded as exploratory and hypothesis-generating rather than predictive. Because the rumen microbiome was not characterized, integrated microbiome and metabolomic analyses are required to validate these hypotheses. The biological conclusions from this study are therefore based primarily on univariate statistical analyses with FDR correction, pathway analysis, and biological interpretation rather than the PLS-DA model, and the identified pathways and biomarkers should be interpreted as candidate biological processes requiring validation through targeted metabolomics, larger independent cohorts, and complementary functional studies.

In conclusion, dietary supplementation with a microfused essential oil blend of oregano, cinnamon, and white thyme improved final body weight, average daily gain, and feed efficiency in Katahdin ewes and was associated with selective alterations in the plasma metabolome, including differentially abundant metabolites, enrichment of three polyunsaturated fatty acid metabolism pathways (linoleic acid metabolism, α-linolenic acid metabolism, and biosynthesis of unsaturated fatty acids), and a four-metabolite candidate biomarker panel that discriminated MEO from CON ewes (combined AUC = 0.943). *γ*-amino-*γ*-cyanobutanoic acid and isoleucine emerged as candidate metabolites independently associated with growth performance and feed efficiency in this exploratory study. However, these associations require validation in larger, independent populations before predictive utility can be established. Together, these results indicate that plasma metabolite signatures identified in this study could signify a promising putative biomarker of MEO supplementation and growth performance in growing ewes. However, these findings are exploratory and require validation through targeted metabolomics, larger independent cohorts, integrated microbiome analyses, and functional studies before they can be translated into practical biomarkers or mechanistic indicators of essential oil supplementation in ruminant.

## Data Availability

The original contributions presented in the study are included in the article/Supplementary material, further inquiries can be directed to the corresponding author.
